# Longer, More Active Commute, but Still not Very Active: Five-Year Physical Activity and Travel Behavior Change in a University Population

**DOI:** 10.3390/ijerph16132420

**Published:** 2019-07-08

**Authors:** Lina Engelen, Erika Bohn-Goldbaum, Melanie Crane, Martin Mackey, Chris Rissel

**Affiliations:** 1 University of Sydney, Sydney School of Public Health, Sydney 2006, Australia; 2 University of Sydney, Faculty of Health Sciences, Lidcombe 2141, Australia; 3 NSW Office of Preventive Health, Liverpool 2171, Australia

**Keywords:** active transport, public transport, physical activity, university students

## Abstract

Active travel can support the achievement of recommended levels of physical activity. Monitoring travel behavior of university students and staff provides a useful insight into patterns of regional travel and population level changes in physical activity. This study sought to evaluate current travel and physical activity behaviors in a university population and to determine whether these changed over time. An online survey of travel behavior and physical activity was conducted at the University of Sydney, Australia. The survey was actively promoted for three weeks prior to the release of the survey among staff and students, which asked about travel behavior on a specific day in September 2017. The survey questions were the same as those used in a similar online survey conducted across the University in 2012. In total, 4359 People completed the survey, representing 10.8% of staff and 4.1% of students. Approximately two thirds of survey respondents were students, in both the 2012 and 2017 surveys. Compared with 2012, there was an increase in active travel to the University in 2017 from increased walking and train travel. Compared to 2012, in 2017 there was an increase in average minutes walked by about nine minutes, and less time spent sitting. Trip lengths increased, with 68% of trips taking longer than 30 min in 2017. The amount of time spent in low–moderate levels physical activity increased between 2012 and 2017, potentially related to active travel behavior. Citywide changes towards a system-wide transport fare structure was the biggest change in the transport environment between the two surveys and may have contributed to increased train travel.

## 1. Introduction

Despite the known benefits of physical activity (PA) and risks associated with inactivity, worldwide almost one quarter of adults are insufficiently active [1]. A lack of sufficient physical activity is one of the leading risk factors for all-cause mortality [2]. Insufficient PA for adults, defined as less than 150 min moderate activity per week, is associated with an increased risk for coronary heart disease, diabetes, stroke, dementia, and some cancers [3]. It is therefore important to monitor PA and sedentary behaviors in order to identify areas in which strategies for better population health might be introduced.

A decline in population active transportation in favor of motor vehicles has been recognized as a key contributor to physical inactivity [4]. Therefore, greater attention has been placed on improving active travel as a recommendation for increasing PA in the population [1]. The journey to work or study is an important trip to target because it is repetitious. Public transport is often included as a mode of active travel because it will generally involve some walking or cycling between destinations.

As is true in other English-speaking countries (e.g., USA, Canada, and UK), the rate of active travel in Australia has declined over time while at the same time obesity levels have increased [5]. Where walking, cycling, and public transport has declined, Mees and Groenhart showed the number of car drivers in Australia rose by 94% between 1976 and 2011 [6]. According to the current 2016 census, the majority (68.4%) of Australians now travel to work or study by car, while 11.5% use public transport and less than 5% walk [7]. In cities like Sydney, public transport use is higher (26.3% in 2016); however, only 4.6% of the population in metropolitan Sydney walk and less than 1% cycle to work.

University populations are valuable for monitoring travel behavior changes over time as they travel to a central area from a wider catchment region, allowing observations on the impact of transport changes on commuting habits and their relation to PA levels to be evaluated. Evidence suggests the actively commuting university populations are healthier than their colleagues. In the USA, active commuting students have better objectively measured cardiovascular fitness, are more flexible, and have lower systolic blood pressure than non-actively commuting students [8]. World-wide, studies have found increased levels of PA among university staff and students who commute to campuses by walking, cycling, or public transport compared to those using private vehicles [9,10]. One study in the state of Western Australia found students who used public transport to commute to university attained more daily walking than students who used a private vehicle (11,443 vs. 10,242 steps/day) [11]. In another study, 46% of students and staff in Sydney who walked or cycled to university attained sufficient weekly PA, compared to just 39% of those using private or public transport [12].

Further, university populations can increase their energy expenditure in response to short-term transport changes. In a four-week study, student bus riders demonstrated an increase in their number of steps during an industrial action by the bus company [13]. The introduction of express transit increased ridership significantly (from 5.5% to 8.5% over one year) among Canadian university staff, and these transit riders had 80 min/week more transport PA and 50 min/week more total PA than passive commuters [9]. Similarly, the introduction of a public bike share program increased bicycle commuting by Spanish university students significantly from 6.9 to 11% after eight months, and those that used a public bike share program expended 257 metabolic equivalent minutes/week more than at baseline [14]. Most recently, an eight-month long intervention using a smartphone app and social marketing increased active travel among American students (from 49.2% to 64.2%), but not among staff (7.9% to 8.91%), compared to the previous year [15].

Unfortunately, long-term studies monitoring travel behavior in university populations have rarely also measured PA. British university staff travel surveys from 1998 to 2007 indicated an increase in walking (19 to 30%) and cycling (7 to 11.8%) as commute modes; further, of those who regularly used active transport, most (70%) achieved almost all (80%) of the recommended PA through their commute [16]. At an Australian university, the percentage of students and staff walking remained consistent (around 12%) from 2007 to 2016, while bicycling increased significantly from 2.7% to 4.1%; however, PA levels were not measured [17]. These studies indicate some change in active travel, but it is uncertain how much PA the student or staff population are attaining from their commute and whether it translates into sufficient activity overall.

In 2012, a survey of staff and students from The University of Sydney revealed 23% of staff/students were travelling to the university via active travel [12]. Since the 2012 survey, a number of transport related changes have occurred in the city; these include government-led changes intended to increase public transport usage (e.g., new system-wide transport fare structure and ongoing expansion of light rail) and steps by the City of Sydney council to develop a network of regional bicycle paths [18]. Further, the university has since introduced changes in travel policy and infrastructure (further described in Methods). A repeat of the survey allows us to observe what impact these changes may have had on transport choices to the university and overall PA. Therefore, the purpose of this study is to evaluate current travel and PA behaviors in a university population and to determine whether these changed over time.

## 2. Materials and Methods

### 2.1. Setting

The University of Sydney has a student population of 67,720 and employs approximately 14,301 faculty and staff. The university’s main campus (at Camperdown) accounts for approximately 86% of the student body and 75.3% of employees. The university is situated 1 km from the nearest metro train station and within 2 km of the central station interchange, connecting suburban, intercity, and regional train services and bus routes; 20 bus routes pass alongside the main university campus. The university has over 12 satellite campuses. Most of these satellite campuses are located within five minute’s walking time from public transport stops. The few satellites (mostly agricultural facilities) not serviced by public transport account for only a very small portion (<0.4%) of the university’s population. The university promotes major walking and cycling events, such as the Australian Ride to Work Day. In and around the university there are a number of end-of-service facilities: a reduced-fee bicycle mechanic works on the main campus; there are bicycle repair and water refill stations and outdoor bicycle racks are available throughout the main and some satellite campuses; and new staff buildings are increasingly including end-of-trip facilities (shower and locker facilities and under cover bicycle storage) in the building design.

### 2.2. Research Design

An online survey was used to collect travel behavior data from all staff and students from the university. The survey was promoted through online staff and student media and flyers three weeks prior to the survey data collection date. On the day the survey was taken, an automated email invitation containing a hyperlink to the survey was sent to all student and staff email accounts. One reminder via Twitter was sent on the survey date. The survey remained open for approximately 24 h post automated invitation. We chose to use the single day ‘census’ style approach to keep the methodology consistent with the original survey from 2012 to facilitate comparisons. Further, a census-based approach will generate an accurate estimate of lower frequency modes or variables when the sample size is sufficiently large, as was the case in this study.

The survey asked respondents about their travel behavior on a specific Tuesday, the 19th September 2017. The weather on the day of the survey was 14.0–25.2 °C and sunny [19], which is within the usual range for that time of year.

Details of the 2012 survey have been published elsewhere [12].

### 2.3. Measures

Measures included in this paper were the same in 2017 as in 2012, except for total walking minutes. Respondents were asked if they travelled to the university on the census day, which campus they travelled to, and the travel mode they used for the longest (by distance) part of the trip. (See Supplementary File for full survey.) Respondents were then asked to indicate if any other additional mode of travel was used or if intercampus travel was undertaken.

Physical activity was measured over the last 7 days using the International Physical Activity Questionnaire short form (IPAQ-SF) [20]. Measures for minutes in vigorous physical activity (VPA), moderate physical activity (MPA), walking for 10 min or more, and sitting were used. Respondents also provided demographic information (i.e., age, sex, educational achievement, residential postcode, and student/staff status).

The research was approved by the Human Research Ethics Committee, The University of Sydney (Protocol No. 2017/623).

### 2.4. Statistical Analysis

Demographics and main outcomes were summarized descriptively. Linear regressions were performed on the outcomes of weekly frequency of walking bouts (of ≥10 min), MPA and VPA, and of weekly sitting time. Year (2012/2017); type of travel (active, public, private); age; gender; role (staff/student); and faculty (health/non-health) were included as factors. Factors that were not significant were removed from the model in a step-wise fashion with the least significant factor removed first, until all remaining factors were significant.

A proportions test was used to test for significant differences in proportions. *P* < 0.05 was considered statistically significant.

## 3. Results

### 3.1. Demographics

In 2017, the university population comprised 75,986 persons; of these 6344 (8.4%) attempted the survey with 4359 (69%) completing it. Staff (1548) and student (2786) respondents represented 10.8% and 4.1% of the university staff and student populations, respectively. Most survey participants (71.9%) were from the university main campus. The largest proportion of the respondents across both the 2017 and 2012 surveys were students (63% in 2017 and 60% in 2012). Professional staff constituted the largest part of the staff sample in both survey years. However, there were some significant differences between years (Table 1). In 2017, 64% of the respondents were female, whereas in the 2012 survey 69% of respondents were female. As would be expected in a university setting, the respondents were largely well-educated, with 65% having a tertiary degree or higher in 2017 while 61% of respondents had tertiary education 2012. The majority (70%) of the 2017 sample was young (<34 years), which was significantly more than in 2012 (65%). There were more female student survey participants (72.2% and 63.9%) compared with enrolled female students at the University (57.3% and 58.2%) in 2012 and 2017, respectively (both *p* < 0.001). There were also more female staff survey participants (63.9% and 64.5%) than on the University payroll (54.8% and 55.0%) in 2012 and 2017, respectively (both *p* < 0.001).

### 3.2. PA and Sitting Time

**Figure 1:**
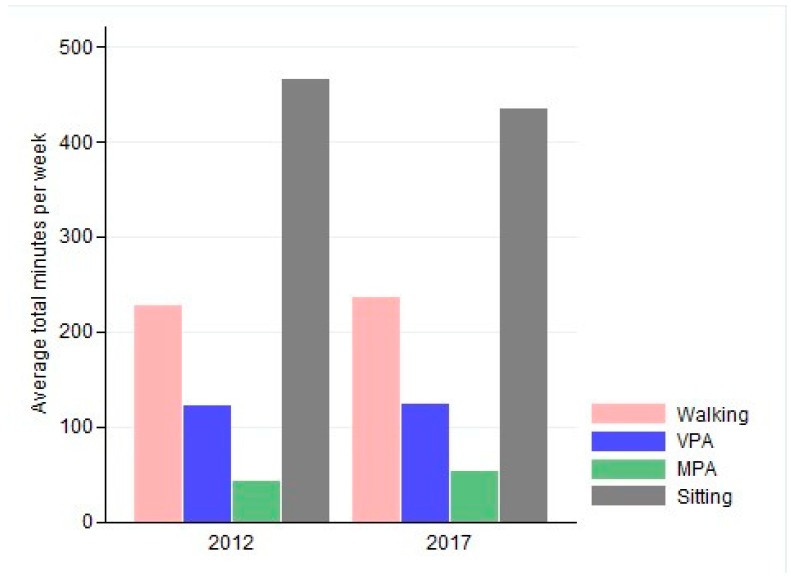
Average weekly minutes of physical activity and sitting time in 2012 and 2017. Walking: minutes of walking in bouts of at least 10 min; VPA: Vigorous physical activity; MPA: Moderate physical activity.

Respondents in the 2017 survey reported engaging in an average weekly 235 min of walking for at least 10 min, 53 min moderate activity, and 124 min vigorous physical activity (Figure 1). Compared to 2012, this was about 9 min more walking, in 10-min bouts per day (*p* = 0.028, 95% CI: 0.98; 17.1), and almost 10 min more moderate physical activity per day (*p* = 0.000, 95% CI: 5.2; 14.6). In addition, respondents in 2017 reported sitting about 32 min less per day than in 2012 (*p* = 0.000, 95% CI: −39.3; −25.2) (Table 2). There was no significant difference between 2012 and 2017 in reported VPA. Reported walking in 10-min bouts was 22 min higher for staff than for students (*p* = 0.000, 95% CI: 8.5; 25.1) and 36 and 81 min higher for those who used active transport to get to the university rather than public or private transport, respectively (*p* = 0.000, 95% CI: −48.5; −24.3 and *p* = 0.000, 95% CI: −93.0; −69.5).

### 3.3. Main Travel Mode

In 2017, the main mode of travel respondents used to access the university was by train, with 35.5% of respondents arriving by this mode. Students were more likely to use train to commute to the university than staff (39.6% vs. 28.6%, respectively, *p* < 0.0001). A few notable changes were found in main travel mode between the 2012 and 2017 travel survey (Table 3). The proportion of respondents that reported walking as their main mode to get to the university on the survey days increased from 17% to 23% (*z* = 11.1, *p* < 0.0001), and traveling by train increased from 32% to 35% (*z* = 9.7, *p* < 0.0001) between 2012 and 2017. Conversely, the proportion traveling by car or motorcycle decreased from 27% to 18% (*z* = 9.6, *p* < 0.0001), respectively, between 2012 and 2017. Changes were also observed in the proportion using walking or cycling modes, which increased from 23% to 29% (*z* = 5.1, *p* < 0.0001). Overall most (82.2%) respondents commuted to the university by active modes (including public transport) and this was higher than in 2012 (72.5%; *p* < 0.000).

### 3.4. Trip Length

The length of time respondents spent getting to campus increased from 2012 to 2017 (Table 4). Fewer respondents commuted to campus in less than 30 min (38% in 2012 vs. 32% in 2017). Most staff and students spent more than 30 min traveling to university (67.9% in 2017). Similarly, the proportion of respondents that spent more than 60 min getting to campus increased from 23% to 30% between 2012 and 2017.

## 4. Discussion

This study investigated changes in physical activity and transport behavior of university staff and students in Sydney over a five-year period. The results of this study reveal some significant changes. Specifically, the amount of time spent in low–moderate level physical activity increased between 2012 and 2017 while sitting time decreased. These trends are encouraging to see from a public health perspective.

Physical activity increases were also reflected in the travel behavior in the population. The findings revealed an increase in active travel and public transport modes and a decrease in passive mode travel in the university population between 2012 and 2017. Many studies have shown how active travel contributes towards total physical activity and overall health [21,22,23,24]. Without knowledge of how participants spent the rest of their day in both surveys, we cannot say that the changes in overall physical activity were directly reflective of changing commuting behavior towards active transport. However, this study shows a strong correlation between those participants achieving sufficient physical activity and the mode of travel, in support of the literature.

Commuting by public transport has been shown to provide sufficient means of achieving on average 15 min of physical activity per trip [25] and can be sufficient for achieving recommended levels of physical activity. The distance between the main university campus and metro train stations is more than 10 min’ walking distance for most staff and students, which means they would be able to achieve a good part of the recommended 30 min’ activity per day from this leg of their commute alone. As students were more likely than staff to use public transport modes and active transport for commuting to study, they may have been more likely to meet this recommendation. This current study has also revealed how commuting times have changed over the past five years, with more staff and students traveling more than 30 min per trip to reach their place of work or study. Long travel times can be a risk to physical and mental health, with some evidence suggesting an increased risk of obesity (in the case of driving) [26], depression, and mental stress [27]. According to recent research, the average Sydney commuting time is currently 37.5 min per trip and up to 63 min for trips into the central business district (CBD) [28]. In accordance with this, with most of the sample travelling to the University’s main campus, located adjacent to the CBD, nearly one third of our study sample in 2017 commuted for more than one hour to reach their work or study. Increasing travel times may have other ramifications, such as reduced social time; however, the evidence is limited [29].

The study findings create a number of questions regarding how these increases in physical activity and active travel may have occurred. In the five years between 2012 and 2017, a number of transport changes have occurred, as mentioned earlier. In addition, we have seen a downward trend in cycling across the city and nationally [30], which may be attributed in part to improvements in public transport, route disruption in the city caused by construction of a new light rail, and removal of one major bicycle path. Walking increased in the study from 16% to 23% over the five years, which may be a result of increased campus parking costs or other transport or urban policy measures, and which requires further exploration. There is a possibility that the differences in demographics between the 2012 and 2017 surveys contribute to the outcomes. In comparison to the 2012 sample, the 2017 sample was younger and better educated and had a larger proportion of male respondents, all of which could contribute to increased physical activity. In line with our findings, however, are the increases in active travel and decreases in driving measured in annual surveys conducted 2007–2016 at another university in Sydney [17].

Our study findings can guide and support city and university policies. For example, the current focus of Sydney’s regional plan is for a 30-min (commute) city [31], which will be important for a number of reasons, including health, urban planning, and sustainability [32]. In a recent study of transport policies in Sydney, the authors found policies to improve walking, cycling, and public transport received high support from Sydney residents irrespective of the mode of travel they use to commute to work or study [33]. This citywide support and the overall increase in active travel and public transport among our university sample suggest that providing Sydney residents more policy and infrastructure support to use active travel modes can be successful. The University of Sydney has a wide catchment area, attracting staff and students from over one hour’s travel distance, and so can indicate how a wider city population may also be changing behaviors. Repeated surveys like this university survey are a good way of exploring the effects of policies and interventions—such as major planned road and public transport changes, the introduction of dockless bicycle share schemes, and the rapid rise of Uber—as they are snapshots of population behaviors. The low response rate as a percentage of the university population is a limitation; however, it is similar in number to the previous survey and the repeated cross-sectional design is a feasible approach to provide a large sample.

The measures used in this study were self-reported. The weaknesses of using self-reported physical activity data have been examined elsewhere [34]. Self-reported data using questions from the IPAQ-SF questionnaire are likely to overestimate how much physical activity people do; it has been reported that the IPAQ-SF overestimates the level of physical activity by on average 84 percent [35,36]. However, the more reliable alternative of objective physical activity monitoring is often not feasible for large scale studies and population surveillance. Although linear regression models like the ones we used are commonly used to evaluate repeat cross-sectional data, there is potential for over/under estimation of effects.

Other potential limitations (e.g., sample size and demographics) have been mentioned above.

## 5. Conclusions

This study provides some important insights into the physical activity and transport behavior changes of a university population in Sydney. While there has been some increase in light–moderate physical activity and active travel, the greater university population is still not very active. Local university and government policies to improve physical activity in the larger population are needed. University populations provide a good place not only for assessing changes but also, given universities’ keen interest in research, a fostering ground for testing and implementing new policies to improve health and sustainable urban transport outcomes.

## Figures and Tables

**Table 1:** Demographic characteristics of the study samples.

		2012 *N* (%)	2017 *N* (%)	Difference between Years (chi^2^, *p*-Value)
Gender	male	1123 (31%)	1561 (36%)	
	female	2490 (69%)	2760 (64%)	
	other		27 (1%)	44.9, *p* = 0.000
Role Type	staff academic	515 (14%)	519 (12%)	
	staff professional	857 (23%)	968 (22%)	
	affiliate	65 (2%)	61 (1%)	
	student undergraduate	1393 (38%)	1624 (37%)	
	student postgraduate	803 (22%)	1162 (26%)	
	other	29 (1%)	25 (1%)	30.13, *p* = 0.000
Education	completed primary	11 (0.3%)	11 (0.3%)	
	completed secondary	1196 (33%)	1357 (31%)	
	diploma	210 (6%)	186 (4%)	
	tertiary	1691 (46%)	2257 (52%)	
	PhD	533 (15%)	548 (13%)	28.75, *p* = 0.000
Age Category	<25	1410 (40%)	2107 (48%)	
	25–34	870 (25%)	941 (22%)	
	35–44	516 (15%)	568 (13%)	
	45–54	413 (12%)	397 (9%)	
	55–64	249 (7%)	256 (6%)	
	65+	59 (2%)	79 (2%)	59.59, *p* = 0.000

**Table 2:** Results of regression analyses on comparisons of sitting, moderate physical activity (MPA), vigorous physical activity (VPA), and walking in 10-min bouts between the years 2012 and 2017.

Variable	Coefficient	T	*p*	95% CI
**Sitting (min) ^a^**	−32.25	−8.97	0.000	−39.3; −25.2
**MPA (min) ^b^**	9.87	4.11	0.000	5.2; 14.6
**VPA (min) ^c^**	−0.78	−0.22	0.83	−7.8; 6.2
**Walking 10 Min (min) ^d^**	9.04	2.20	0.028	0.98; 17.1

Type of travel (active, public, private); age; gender; role type (staff/student); faculty (health/non-health); and year (2012/2017) were included as factors. Factors that were not significant were removed from the model in a step-wise fashion with the least significant factor removed first, until all remaining factors were significant. Final models include the following significant factors: a. Year; b. Year; c. None (no significant factors); d. Year, role type.

**Table 3:** Reported main mode of travel to university on travel survey day in 2012 and 2017.

Main Mode of Travel to Uni	Year			
	2012		2017	
	*n*	%	*n*	%
Walk	500	16.6	865	23.0
Bicycle/skateboard/scooter	194	6.4	212	5.6
Train/light rail	954	31.6	1336	35.5
Bus	515	17.1	640	17.0
Car/motorcycle	825	26.7	665	17.5
Other *	28	0.9	45	1.2
Total	3016		3763	
	Pearson chi^2^ (12) = 127.61; *p* = 0.000			

* Other refers to modes that do not fit into the other categories, such as airplane, ferry, or a combination of equal time in several modes of transport; hence, it was not possible to place them in either category.

**Table 4:** Trip length in minutes in 2012 and 2017.

Trip Length (min)	Year			
	2012		2017	
	*n*	%	*n*	%
0–9	103	3.57	110	3.01
10–19	491	17.04	528	14.44
20–29	506	17.56	537	14.69
30–39	421	14.61	592	16.19
40–49	334	11.59	548	14.99
50–59	343	11.9	258	7.06
60–74	333	11.55	486	13.29
75–89	121	4.2	180	4.92
90–300	215	7.46	417	11.41
Total	2882		3656	
	Pearson chi^2^ (9) = 125.14 *p* = 0.000			

## Supplementary Materials

The following are available online at https://www.mdpi.com/1660-4601/16/13/2420/s1, full survey.
