# Supplementary material for: Longer, More Active Commute, but Still not Very Active: Five-Year Physical Activity and Travel Behavior Change in a University Population
Source: Int J Environ Res Public Health. 2019 Jul 8;16(13):2420. doi: 10.3390/ijerph16132420 (PMC6651375; doi:10.3390/ijerph16132420)
Supplement: Supplementary file 1 [file ijerph-16-02420-s001.pdf]

Welcome

Welcome to the 2017 University of Sydney Travel Census

The University is conducting a survey of physical activity and travel by staff and students to our campuses on 19 September 2017. This information will help inform strategies for the 'move more, sit less' component of the Healthy Sydney University initiative.

The full participation information statement, prize information and more information about the census can be accessed here. Briefly,

- Participation is voluntary.
- The survey is designed to take less than 10 minutes and participation can be anonymous.
- To thank you for your time, you can win one of several prizes.
- A summary of results will be publicly available.
- Contact Healthy Sydney University at healthy.uni@sydney.edu.au for further information.

* 1. To continue, please select the 'I wish to continue' box below.

- ◯ Yes, I wish to continue and I voluntarily consent to this participation.
- ◯ No thanks, I wish to exit.

Section 1: Your physical activity

The first question is an overview of your physical activity; this is followed by a few more specific questions.

All of these questions are about your physical activity over the past SEVEN days.

* 2. In the past week, on how many days have you done a total of 30 minutes or more of physical activity which was enough to raise your breathing rate? This may include sport, exercise, brisk walking or cycling for recreation or to get to and from places, but should not include housework or physical activity that may be part of your job.

days per week [ ]

* 3. In the last week, how many times have you walked continuously, *for at least 10 minutes*, for recreation, exercise or to get to or from places?

[ ]

* 4. What do you estimate was the total time that you spent walking *in this way* in the last week?

hours

[ ]

AND minutes

[ ]

* 5. In the last week, what do you estimate was the*total* time that you spent walking?

hours

AND minutes

* 6. In the last week, how many times did you do any vigorous physical activity which made you breathe harder or puff and pant (e.g., jogging, cycling, aerobics or competitive tennis)?

times

* 7. What do you estimate was the total time that you spent doing this vigorous activity in the last week?

hours

AND minutes

* 8. In the last week, how many times did you do any other more moderate physical activity that you have not already mentioned (e.g., gentle swimming, social tennis or golf)?

times

* 9. What do you estimate was the total time that you spent doing this moderate activity in the last week?

hours

AND minutes

* 10. In the last week, how much time did you usually spend sitting on a weekday?

hours

AND minutes

Section 2: Your travel on Tuesday, 19 September

This section asks about your travel to a University of Sydney campus on Tuesday, 19 September. If you travelled to more than one University of Sydney campus on this day, please answer these questions in regards to the FIRST campus you visited. You will have an opportunity to tell us about visits to other campuses in a later section of the census.

* 11. To which University of Sydney campus did you travel on Tuesday, 19 September? (Tick first campus visited.)

- ◯ I was not on any University of Sydney campus that day.
- ◯ Australian Technology Park
- ◯ Broken Hill
- ◯ Camden Campus
- ◯ Camperdown/Darlington Campus (including Burren St and Mallett St Campuses)
- ◯ Conservatorium Campus (Sydney Conservatorium of Music)
- ◯ Cumberland Campus (Faculty of Health Sciences)
- ◯ Dubbo
- ◯ Narrabri
- ◯ Orange
- ◯ Rozelle Campus (Sydney College of the Arts)
- ◯ Surry Hills Campus (Faculty of Dentistry)
- ◯ Sydney Medical School Campuses
- ◯ Westmead Precinct
- ◯ Other (please specify)

Camperdown area campus visitors

This question asks about your destination on the Camperdown-Darlington campus. By 'first destination' we mean the first place on campus you stayed for a period of time, such as your office, a lecture theatre, the library, or a laboratory. If you stopped en route, for example, to purchase a coffee, do NOT count that place as your first destination.

* 12. Where was your first destination on campus? Select the destination from the alphabetical dropdown list.

Travel mode

This next question asks about how you travelled to a University of Sydney campus on Tuesday, 19 September. If you used more than one type of transport, please select the method which covered the most distance. You will have an opportunity to tell us about other transport types in a later section of the census.

* 13. How did you travel to campus on Tuesday? Select the mode used for the longest part, by distance, of your journey.

- ◯ Walk
- ◯ Bicycle
- ◯ Skateboard or scooter (non-motorized)
- ◯ Train
- ◯ Bus
- ◯ Light rail
- ◯ Ferry
- ◯ Car as driver
- ◯ Motorcycle as driver
- ◯ Car or motorcycle as passenger
- ◯ Taxi
- ◯ Uber or other paid private transport
- ◯ Other (please specify)

Motorists

* 14. How many passengers were in your vehicle when you arrived at campus today?

* 15. What is your main reason for using the car or motorcycle to get to campus?

- ◯ Dropping/collecting children
- ◯ Dropping/collecting other persons
- ◯ Health reasons
- ◯ Lack of an alternative
- ◯ Convenience
- ◯ Safety
- ◯ Time
- ◯ Cost
- ◯ I'm required to travel between campuses as part of my job/studies
- ◯ I work/study outside of normal business hours
- ◯ Other (please specify)

* 16. Where did you park your vehicle today?

- ◯ On campus - using a paid parking permit
- ◯ On campus - using RTA mobility parking permit
- ◯ On campus - casual paid parking
- ◯ On campus - free
- ◯ Off campus - paid spot
- ◯ Off campus - free
- ◯ Other (please specify)

Vehicle passengers

* 17. How many passengers were in the vehicle when you arrived at campus today? Please answer according to your mode of travel, leaving the other mode blank.

Car passenger

Motorcycle passenger

* 18. What is your main reason for being driven to campus?

- ◯ Health reasons
- ◯ Lack of an alternative
- ◯ Convenience
- ◯ Safety
- ◯ Time
- ◯ Cost
- ◯ I work/study outside of normal business hours
- ◯ Other (please specify)

* 19. Where did the vehicle in which you rode park today after you disembarked?

- ◯ I don't know
- ◯ On campus - using a paid parking permit
- ◯ On campus - using RTA mobility parking permit
- ◯ On campus - casual paid parking
- ◯ On campus - free
- ◯ Off campus - paid spot
- ◯ Off campus - free
- ◯ Other (please specify)

Bus users

* 20. What bus route did you use to get to campus today? Type the route number, e.g., 422. If more than one bus was used, type the last route used.

* 21. How many buses did you use to get to campus?

Train users

* 22. At which station did you get disembark to get to campus today?

- ◯ Redfern
- ◯ Central
- ◯ City - Town Hall, Wynyard, Circular Quay, Museum, St James or Martin Place
- ◯ Lidcombe
- ◯ Macdonaldtown
- ◯ Newtown
- ◯ St Leonards
- ◯ Westmead
- ◯ Other (please specify)

Bicyclists

* 23. Where did you park your bicycle?

- ◯ In an outdoor bicycle rack
- ◯ In other space outside
- ◯ In a secure indoor bicycle storage area
- ◯ Inside an office or work space
- ◯ Other (please specify)

Other travel modes and destinations

* 24. Did you use any other transport mode to travel to campus(es) on Tuesday, 19 September? Select all applicable answers.

- [ ] I did not use another mode of transport
- [ ] Walk
- [ ] Bicycle
- [ ] Skateboard or scooter (non-motorized)
- [ ] Train
- [ ] Bus
- [ ] Light rail
- [ ] Ferry
- [ ] Car as driver
- [ ] Motorcycle as driver
- [ ] Car or motorcycle as passenger
- [ ] Taxi
- [ ] Uber or other paid private transport
- [ ] Other (please specify)

* 25. To which other University of Sydney campuses did you travel on Tuesday, 19 September? (Tick all visited.)

- [ ] I did not visit any other University of Sydney campus that day.
- [ ] Australian Technology Park
- [ ] Broken Hill
- [ ] Camden Campus
- [ ] Camperdown/Darlington Campus (including Burren St and Mallett St Campuses)
- [ ] Conservatorium Campus (Sydney Conservatorium of Music)
- [ ] Cumberland Campus (Faculty of Health Sciences)
- [ ] Dubbo
- [ ] Orange
- [ ] Narrabri
- [ ] Rozelle Campus (Sydney College of the Arts)
- [ ] Surry Hills Campus (Faculty of Dentistry)
- [ ] Sydney Medical School Campuses
- [ ] Westmead Precinct
- [ ] Other (please specify)

Trip length and timing

* 26. How long, in minutes, was your trip from your home to campus on Tuesday, 19 September? (If you visited multiple campuses, please answer in regards to the first campus you visited.)

* 27. At what time did you (or will you) arrive at and depart from campus?

Arrive

hh mm -

Depart

hh mm -

* 28. On which days of the week do you usually travel to a University of Sydney campus during this semester? (Tick all that apply.)

- [ ] All 5 working days (Monday-Friday)
- [ ] All 7 days (Monday-Sunday)
- [ ] Monday
- [ ] Tuesday
- [ ] Wednesday
- [ ] Thursday
- [ ] Friday
- [ ] Saturday
- [ ] Sunday
- [ ] I am not usually on a campus this semester

Section 3: Infrastructure

29. Which of the following changes or initiatives would you like to see on your campus? (Select all that apply.)

- [ ] Improved bicycle paths on the journey to campus
- [ ] Improved bicycle paths on the journey within campus
- [ ] More outdoor bicycle parking on campus
- [ ] More undercover bicycle parking on campus
- [ ] More indoor secure bicycle parking on campus
- [ ] Improved changing facilities & lockers on campus
- [ ] Arrangements to buy a bicycle at a discount
- [ ] Access to ovals for walking during work/study breaks
- [ ] Access to ovals for recreation
- [ ] More shade on campus
- [ ] Better lighting on campus
- [ ] Help in finding walking groups
- [ ] Help in finding a carpool
- [ ] Reserved parking for carpools
- [ ] Improved signposting of walking and cycling paths
- [ ] A bike share or loan scheme
- [ ] Financial incentives or subsidies for public transport
- [ ] Improved foot paths on the journey to campus
- [ ] Improved foot paths within campus
- [ ] Separate walking and bicycling paths
- [ ] Subsidies for gym memberships
- [ ] Maps and advice on how to best walk/bicycle to campus
- [ ] Other (please specify)

30. Which of the following changes would encourage you to use public transport for your journey to campus more often? (Please tick all that apply.)

- [ ] More direct bus routes
- [ ] More frequent bus service
- [ ] Better lighting at bus shelters
- [ ] Better lighting on campus footpaths
- [ ] More convenient bus drop off points
- [ ] Better connection to campus from the station
- [ ] Better connection from home to the station
- [ ] Improved public transport information
- [ ] More secure cycling racks at train stations
- [ ] None, I use public transport for my journey to campus and am satisfied
- [ ] Other (please specify)

Section 4: About you

* 31. Overall, how would you rate your health during the past four weeks?

- ( ) Excellent
- ( ) Very Good
- ( ) Good
- ( ) Fair
- ( ) Poor
- ( ) Very Poor

* 32. Have you ever used a bicycle share program (e.g., Reddy Go or Airbike)?

- ( ) Yes
- ( ) No

About you

* 33. How often do you use a bike share program?

- Every day
- 5-6 days per week
- 1-2 days per week
- 1-2 days per month
- Less than once a month
- Never

* 34. Do you have a disability that affects your choice of travel mode?

- Yes
- No

* 35. In what postcode do you live?

* 36. What is the level of the highest qualification you have completed?

- Completed primary school
- Completed HSC/ Leaving/ Year 12/ 6th form
- TAFE (certificate or diploma)
- University, CAE or tertiary institute degree
- PhD

* 37. With which faculty or unit are you primarily associated?

- Agriculture, Food and Natural Resources
- Architecture, Design and Planning
- Arts and Social Sciences
- Business School
- Dentistry
- Education and Social Work
- Engineering and Information Technologies
- Health Sciences
- Pharmacy
- Science
- Sydney College of the Arts
- Sydney Conservatorium of Music
- Sydney Law School
- Sydney Medical School
- Sydney Nursing School
- University-wide administration or services (eg Library, CIS, ICT, Finance, HR etc)
- Veterinary Science
- Other (please specify)

* 38. What is your role at the University?

- Staff- academic
- Staff- professional
- Affiliate (e.g., honorary, contractor, or volunteer)
- Student- undergraduate
- Student- postgraduate coursework
- Student- postgraduate research
- Other

Students

* 39. Do you live on campus as a residential student?

- ◯ Yes
- ◯ No

* 40. What type of student are you?

- ◯ Domestic student
- ◯ International student

About you

* 41. In your role at the University, are you currently full- or part-time?

- ◯ Full-time
- ◯ Part-time
- ◯ Casual

* 42. What is your gender?

- ◯ Male
- ◯ Female
- ◯ Other (please specify)

* 43. What is your age in years?

Section 5: Comments and end

44. Please add any comments you wish to share about your travel experience.

As a thank you for your time, you are eligible to enter a prize draw. As detailed in here, you are eligible to enter a prize draw- this is a random drawing for one several prizes. Winners are notified by email and have 5 business days from time of notification to collect their prize. By providing your email address you are agreeing to the terms and conditions of the prize draw.

45. Are you interested in participating in the prize draw?

- ◯ I do not wish to be entered in the prize draw.
- ◯ Yes. My email address to use as a prize draw entry is

Thank you for completing the survey!

A summary of results will be publicly available.

Thank you to our prize donors, Sydney Uni Sport & Fitness and University of Sydney Union!
